# The duality of spatial death–birth and birth–death processes and limitations of the isothermal theorem

**DOI:** 10.1098/rsos.140465

**Published:** 2015-04-29

**Authors:** Kamran Kaveh, Natalia L. Komarova, Mohammad Kohandel

**Affiliations:** 1 Department of Applied Mathematics, University of Waterloo, Waterloo, Ontario, Canada N2L 3G1; 2 Department of Mathematics, University of California Irvine, Irvine, CA 92697, USA; 3 Department of Ecology and Evolutionary Biology, University of California Irvine, Irvine, CA 92697, USA

**Keywords:** evolutionary dynamics, stochastic process, numerical simulations

## Abstract

Evolutionary models on graphs, as an extension of the Moran process, have two major implementations: birth–death (BD) models (or the invasion process) and death–birth (DB) models (or voter models). The isothermal theorem states that the fixation probability of mutants in a large group of graph structures (known as isothermal graphs, which include regular graphs) coincides with that for the mixed population. This result has been proved by Lieberman *et al.* (2005 *Nature* 433, 312–316. (doi:10.1038/nature03204)) in the case of BD processes, where mutants differ from the wild-types by their birth rate (and not by their death rate). In this paper, we discuss to what extent the isothermal theorem can be formulated for DB processes, proving that it only holds for mutants that differ from the wild-type by their death rate (and not by their birth rate). For more general BD and DB processes with arbitrary birth and death rates of mutants, we show that the fixation probabilities of mutants are different from those obtained in the mass-action populations. We focus on spatial lattices and show that the difference between BD and DB processes on one- and two-dimensional lattices is non-small even for large population sizes. We support these results with a generating function approach that can be generalized to arbitrary graph structures. Finally, we discuss several biological applications of the results.

## 2. Introduction

Exploring the effect of spatial structure of an evolutionary system and its importance on the dynamics of selection has long been of interest in population genetics [1]. The original stepping stone model developed by Kimura & Weiss [2] is the backbone of the subsequent implementations of spatial structure into evolutionary modelling. Maruyama analysed the fixation behaviour of a Moran process in a geographically structured population and was able to show that regular spatial structures do not enhance or suppress selection [3,4]. More recently, Liberman *et al.* [5] revisited the problem and extended previous models to an arbitrary graph, where at each node of the graph a single species can reside. They were able to show that some categories of graphs (such as a star graph, where a central vertex is connected to all leaf vertices) enhance the selection, while other graphs suppress the selection (see also [6,7]). Masuda & Ohtsuki [8] considered the evolutionary dynamics in directed networks and studied the fixation probability of a mutant in finite populations for several update rules. The effects of spatial structure have been also discussed in the spatial evolutionary games (e.g. [9–11]). Other notable recent works have focused on the effect of spatial structure on evolution of cooperation [12] and on the replicator dynamics on a graph structure [13]. Selection dynamics on various types of random graphs has been the subject of a lot of interest recently in the context of applications to social networks [14], infectious disease and epidemiology [15,16], and cancer modelling [17,18], among others. Multi-hit processes and tumour suppressor gene inactivation [19], spatial pattern formation in evolutionary models [20,21] and the effect of spatial distribution of fitness on selection [22] are some other directions of current research. For a general review of evolutionary dynamics on graphs and some recent trends, see Shakarian *et al.* [23].

In the Moran process, we assume the existence of *N* individuals. To keep the population constant, it is assumed that each elementary update consists of a birth and a death event, where individuals are chosen for birth and death from the pool consisting of the entire population. It is of little consequence whether the first event in each elementary update is a birth or a death event. However, the order of these events can become important if we consider a spatial generalization of the Moran process.

To this end, we consider a regular spatial lattice or an unstructured mesh, and again impose the condition of a constant population. For each update, an independent birth (or death) event can occur at a randomly chosen site of the lattice, but the subsequent death (or birth) event should now happen at a neighbouring site of the lattice to represent the spatial structure and the fact that only daughters of individuals that are connected to a particular site can be placed there. In a birth–death (BD) process, a birth event is followed by a death event, and in a death–birth (DB) process, a death event happens first. These are the two main implementations of the generalized Moran process that have been considered in the literature.

Aside from DB or BD models, there have been other implementations of evolutionary processes on a graph structure. Sood *et al.* [14,24] discuss a model of link dynamics, where instead of random sampling of nodes, one considers random samples edges (links) of a graph as a first elementary event and then updates the values of the two nodes on the chosen link based on the fitness of individuals there (similar models are discussed in [13,25]). Evolutionary games in the context of linking dynamics have also been studied [26–28]. Another variation of BD models on a graph is considered by van Baalen & Rand [29], where each of the elementary events can be either a birth or a death or a migration (see also [18,30]).

It has been observed by Maruyama [3,4] and later generalized by Lieberman *et al*. [5] that in the case of the BD process, selection dynamics are not affected by regular and symmetric structures (or more generally isothermal graphs). This elegant result is referred to as the isothermal theorem. In this paper, we explore the applicability of this theorem to other types of processes. The motivation for this question is the result of Komarova [17], where the Moran process was studied on a one-dimensional spatial lattice, and the DB formulation was used. It was shown that the probability of mutant fixation in this case was different from the one obtained in the space-free Moran process. Therefore, in this paper, we study the connection between DB and BD processes on spatial lattices and explore the extent to which the order of birth and death events influences the probability of mutant fixation.

In the literature, differences between DB and BD updates have been described in somewhat different contexts. In papers by Fu *et al.* [31] and Ohtsuki & Nowak [32], evolutionary games were studied on graphs, and different behaviour under DB and BD models has been emphasized. In Ohtsuki & Nowak [13], the replicator equation on graphs is studied in the context of DB, BD and ‘imitation’ dynamics; applications include evolutionary games such as Prisoner's Dilemma, the Snow-Drift game, a coordination game, and the Rock–Scissors–Paper game. Zukewich *et al.* [33] studied evolution of cooperation and formulated the corresponding Moran process under the DB and BD implementations. It is shown that for DB updates, cooperation may be favoured in structured populations, while with BD updates this never is. The authors propose a mixed rule where in each time-step DB or BD updates are used with fixed probabilities, and they further derive the conditions for selection favouring cooperation under the mixed rule for various social dilemmas. Our work adds to these investigations by studying the difference between DB and BD updates in the Moran process in the context of mutant fixation.

Another natural generalization of the Moran process that we focus on here concerns the definition of fitness. In the conventional Moran model, all individuals are characterized by their ‘fitness’ parameters. Usually, an individual is chosen for division with a probability weighted by its fitness, and an individual is chosen for death with the probability 1/*N* (such that all individuals are equally likely to be chosen for death, e.g. [5,33]). In a more general setting, however, fitness could be influenced by the death rate as much as it is by the division rate. For example, a cancer cell in tissue could differ from the surrounding cells by its division rate and/or by its death rate. Therefore, in this paper, we aim to explore how the division and the death rates both influence the fixation dynamics of mutants in the DB and BD processes.

The rest of this paper is organized as follows. In §3, we formulate DB and BD models on a graph, with general death and birth rates. We show that DB and BD transition probabilities can be transformed into each other by using a duality property of the two models. To study the differences between the DB and BD processes, we start with the case of a complete graph (where all vertices are connected, §4) and derive closed algebraic forms for fixation probability in either of cases. In particular, we show that the difference between DB and BD implementation on a complete graph vanishes as 1/*N*, as the system size goes to infinity. In §5, we discuss exact solutions of DB and BD models on a one-dimensional circle and show that the differences are no longer negligible even for large system sizes. In §6, we find an approximation for the fixation probability in higher dimensions and the higher connectivities. Based on the results obtained, we conjecture that the difference between the two models has the order of the inverse of the degree of connectivity of the graph. This is supported by the exact solutions in the complete graph and one-dimensional cases, and numerical simulations in the two-dimensional case. We show that our approximation in two dimensions for a regular lattice matches the exact stochastic simulations within a very good error margin. Finally, a discussion is presented in §7.

## 3. Death–birth and birth–death processes on a graph

Assume two species, A (normal, or wild-type) and B (mutant), with corresponding proliferation rates *r*_B_ and *r*_A_ and death rates *d*_B_ and *d*_A_. A general DB process is defined as follows: at each time step, one cell at site *i* (either A or B) is randomly chosen to die with weight *d*_A_ or *d*_B_. One of the neighbouring sites to *i* (denoted by *j*) is chosen at random, with weight *r*_A_ or *r*_B_ to proliferate, and probability *w*_*ij*_ for the offspring to be placed in site *i* (to replace the dead cell). For each site *i*, we set *n*_*i*_=1 if the resident cell is mutant, and *n*_*i*_=0 if the resident cell is wild-type. Thus, the distribution of mutant cells at sites *i* is *n*_*i*_ and the distribution of normal cells is 1−*n*_*i*_. Given the vector ***n***=(*n*_1_,…,*n*_*N*_), the transition probabilities can be written as

3.1 $$\left.\begin{array}{rl} W_{\mathrm{DB}}^{+}(\{n_{i}\}) & =\frac{d_{A}(1-n_{i})}{d_{B}\sum_{k}n_{k}+d_{A}\sum_{k}(1-n_{k})}\frac{r_{B}\sum_{j}w_{ij}n_{j}}{(r_{B}-r_{A})\sum_{l}w_{il}n_{l}+r_{A}}\\ \;\text{and}\;\;W_{\mathrm{DB}}^{-}(\{n_{i}\}) & =\frac{d_{B}n_{i}}{d_{B}\sum_{k}n_{k}+d_{A}\sum_{k}(1-n_{k})}\frac{r_{A}\sum_{j}w_{ij}(1-n_{j})}{(r_{B}-r_{A})\sum_{l}w_{il}n_{l}+r_{A}}. \end{array}\right\}$$

Here, $W_{\mathrm{DB}}^{+}$ (*W*^−^_DB_) stands for the probability that in the DB process, a new mutant (a new wild-type cell) appears at site *i* after one elementary update. Similarly, in a BD process the sequence of the two death and birth events is switched and can formally be obtained by switching *r*'s and *d*'s in equation (3.1), and also $w_{ij}\to w_{ji}$.

3.2 $$\left.\begin{array}{rl} W_{\mathrm{BD}}^{+}(\{n_{i}\}) & =\frac{r_{B}n_{i}}{r_{B}\sum_{k}n_{k}+r_{A}\sum_{k}(1-n_{k})}\frac{d_{A}\sum_{j}w_{ji}(1-n_{j})}{(d_{B}-d_{A})\sum_{l}w_{li}n_{l}+d_{A}}\\ \;\text{and}\;\;W_{\mathrm{BD}}^{-}(\{n_{i}\}) & =\frac{r_{A}(1-n_{i})}{r_{B}\sum_{k}n_{k}+r_{A}\sum_{k}(1-n_{k})}\frac{d_{B}\sum_{j}w_{ji}n_{j}}{(d_{B}-d_{A})\sum_{l}w_{li}n_{l}+d_{A}}. \end{array}\right\}$$

In the case of BD, $W_{\mathrm{BD}}^{+}(\{n_{i}\})$ indicates that a new mutant appears at any site *j* neighbouring site *i*. Note that in the case of a DB process, the death event is a *global* event and the individual is chosen for death among all the individuals on the graph with weight of *d*_A_ or *d*_B_. The following birth event, however, takes places among only the individuals that are connected to the previously chosen individual, and thus constitutes a *local* event. Even though both of the events occur through random sampling with weighted biases *d*_*i*_ or *r*_*i*_, with *i*=A,B, the subset of random sampling is smaller for the second event unless the graph is a complete graph. Similarly, in a BD process, the global event is a birth event, while the death event takes place for the neighbours of the chosen individual on the graph and thus is a local event.

There are specific cases of the above processes that have been often discussed in the literature. The case of *d*_A_=*d*_B_=1 has been typically implemented as either a DB or BD process on a graph. As the reader can easily see, the opposite limit where *r*_A_=*r*_B_=1 while *d*_A,B_ are arbitrary, also leads to equivalent models while the dynamics of the two models are reversed. In other words, a DB model with *r*_A_=*r*_B_=1 is equivalent to a BD model with *d*_A_=*d*_B_=1 and vice versa.

In the case of BD models with *d*_A_=*d*_B_, it has been argued that for a wide range of graph structures, known as isothermal graphs, where $\sum_{j}w_{ij}=\sum_{i}w_{ij}=1$, the fixation probability of the model is the same as the mixed population one [5] (see also [3,4,7]). The main examples of isothermal graphs are regular lattices with periodic boundary conditions, and the introduction of boundaries or randomness in connectivities removes the isothermal property (for effect of boundaries see [6,17,18]).

## 4. Fixation probability for death–birth and birth–death processes on a complete graph

Let us consider the simplest process which is on a complete graph, where every two nodes *i* and *j* are connected, i.e. *w*_*ij*_=1/*N*. Let us use the following convenient notation:

$$\frac{r_{B}}{r_{A}}=r,\;\frac{d_{B}}{d_{A}}=d.$$

The Kolmogorov backward equation for the absorption probability *π*_*m*_, where *m* is the mutant population, is written as

4.1 $$\left.\begin{array}{rl} \pi_{m} & =W_{m+1}^{+}\pi_{m+1}+W_{m-1}^{-}\pi_{m-1}+(1-W_{m}^{+}-W_{m}^{-})\pi_{m}\\ \;\text{and}\;\;\pi_{0} & =0,\;\pi_{N}=1, \end{array}\right\}$$

which is valid for both BD and DB processes. Transition probabilities $W_{m}^{\pm }$ are defined as the probabilities to gain or lose one mutant in a system with *m* mutants. The general solution for the fixation probability starting from only one mutant can be found in closed form. Denoting

$$\gamma_{m}=\frac{W_{m}^{-}}{W_{m}^{+}},$$

we obtain for the probability of fixation,

4.2 $$\pi_{1}=\frac{1}{1+\sum_{j=1}^{N-1}\gamma_{1}\cdots \gamma_{j}}.$$

Note that the above result is true when the transition probabilities in such a one step process only depend on the number of mutants in the system at every time step, and not on other degrees of freedom of the system. This condition holds for the complete graph, and, as we will see later, for one-dimensional rings.

Next, we will consider some implementations of DB and BD processes on a complete graph. First, we will assume the update rules where the second event (death in the BD process and division in the DB process) occurs in a neighbourhood of a given cell, which includes the cell itself. That is, for example, if a cell is chosen for reproduction first, during the death event this (mother) cell will be in a pool of cells that have a probability to die. This is equivalent to having a graph on which every node is connected to itself by a loop. (Ohtsuki & Nowak [12] call this an ‘imitation’ updating in the context of game theory on graphs.)

Later in this section, we will include a process where the neighbourhood does not includes ‘self’ as a candidate for the second event to take place. The preference for either of the two updatings is somewhat arbitrary and depends on the particular nature of the modelling problem at hand.

### 4.1 The complete network including ‘self’

First, we will consider the process where the network of neighbours of a given cell includes the cell itself. To explain the update procedure in more detail, note that at every time step, we randomly *label* a cell for death (birth) and then label a cell for birth (death). We let the death and birth events happen after both labels have been assigned. This way, for the complete graph (mass-action) scenario, the number of ‘neighbours’ is always *N*, and we have the following transition probabilities:

4.3 $$\left.\begin{array}{rl} W_{m}^{+} & =\frac{rm}{rm+N-m}\cdot \frac{N-m}{dm+N-m}\\ \;\text{and}\;\;W_{m}^{-} & =\frac{N-m}{rm+N-m}\cdot \frac{dm}{dm+N-m}. \end{array}\right\}$$

Interestingly, these probabilities are the same for the DB and BD processes, the two are completely equivalent in this case. We therefore have

$$\gamma_{m}=\frac{d}{r},$$

and the following well-known result for the fixation probability holds:

4.4 $$\pi_{1}=\frac{1-d/r}{1-{\left(d/r\right)}^{N}}.$$

Next, we will study networks that do not include ‘self’. For example, in the BD process described below, after the initial birth event, the cell that has just divided is excluded from the death event. In the following section, for the DB process, the cell that has been chosen for death will not be participating in the subsequent reproduction event.

### 4.2 The birth–death process

For a BD process on a complete graph excluding ‘self’, the transition probabilities $W_{m}^{\pm }$ are written as

4.5 $$\left.\begin{array}{rl} W_{\mathrm{BD},m}^{+} & =\frac{rm}{rm+N-m}\cdot \frac{N-m}{dm+N-m-d}\\ \;\text{and}\;\;W_{\mathrm{BD},m}^{-} & =\frac{N-m}{rm+N-m}\cdot \frac{dm}{dm+N-m-1}. \end{array}\right\}$$

For *d*=1 the ratio of $W_{\mathrm{BD},m}^{-}/W_{\mathrm{BD},m}^{+}=1/r$ for any *m*; however, this is not in general true as upon choosing a mutant/normal cell to divide (birth event) the next death event among the rest of the *N*−1 cells will normalize differently in *W*^−^ and in *W*^+^. In other words, when a mutant cell is chosen to divide (*W*^+^ contribution) the successive death event happens among the rest of the *m*−1 mutants (excluding the one already chosen to divide) and *N*−*m* existing normal cells. When a normal cell is chosen to divide (*W*^−^ contribution), the successive death event occurs among *m* mutant and *N*−*m*−1 normal cells. In general, the ratio of $W_{\mathrm{BD},m}^{-}/W_{\mathrm{BD},m}^{+}$ is

4.6 $$\gamma_{m}^{\mathrm{BD}}\equiv \frac{W_{\mathrm{BD},m}^{-}}{W_{\mathrm{BD},m}^{+}}=\frac{d}{r}\times \left(1-\frac{d-1}{N-m+dm-1}\right).$$

For *d*=1, the ratio will be 1/*r* and substituting into equation (4.2) give rise to the well-known Moran result, equation (4.4),

4.7 $$\pi_{1}=\frac{1-1/r}{1-{\left(1/r\right)}^{N}}.$$

For general values of *d*, however, a closed form for *π*_1_ can be obtained,

$$\pi_{1}^{\mathrm{BD}}=\frac{(d-1)r}{d(N-1)\Phi (d/r,1,(N+d-2)/(d-1))+r{\left(d-1-(N-1)(d/r\right)}^{N}\Phi (d/r,1,(N+d-2)/(d-1)))},$$

where the Lerch transcendent is defined as

$$\Phi (z,s,a)=\sum_{k=0}^{\infty }\frac{z^{k}}{{\left(a+k\right)}^{s}}.$$

To evaluate the large-*N* behaviour of the fixation probability for advantageous mutants, we note that the function *Φ*(*d*/*r*,1,(*N*+*d*−2)/(*d*−1)) decays as a power law as N→∞, and (*d*/*r*)^*N*^ decays exponentially for *d*<*r*. Therefore, we can ignore the term multiplying (*d*/*r*)^*N*^. Further, we note the following asymptotic behaviour of the Lerch transcendent:

$$\Phi (z,1,a)=\sum_{k=0}^{\infty }\left(\frac{z^{k}}{a}+\frac{kz^{k}}{a^{2}}\right)+O\left(\frac{1}{a^{3}}\right)=\frac{1}{a(1-z)}+\frac{z}{a^{2}{\left(1-z\right)}^{2}}+O\left(\frac{1}{a^{3}}\right).$$

Substituting this expression with *z*=*d*/*r* and *a*=(*N*+*d*−2)/(*d*−1) into the equation for *π*_1_, and expanding further in Taylor series in 1/*N*, we obtain

4.8 $$\pi_{1}^{\mathrm{BD}}=1-\frac{d}{r}+\frac{(d-1)d}{rN}+O\left(\frac{1}{N^{2}}\right).$$

The same expansion can also be obtained by a different method. If we expand the expression for $\gamma_{m}^{\mathrm{BD}}$, equation (4.6), in terms of small 1/*N*, we obtain

4.9 $$\gamma_{m}^{\mathrm{BD}}=\frac{d}{r}-\frac{(d-1)d}{rN}+O\left(\frac{1}{N^{2}}\right),$$

an expression that does not depend on *m*. This means that up to the order *O*(1/*N*) we can simply use the geometric progression formula and obtain the result presented in equation (4.8).

### 4.3 The death–birth process

Similarly, for a DB process on a complete graph, the transition probabilities $W_{m}^{\pm }$ are given by

4.10 $$\left.\begin{array}{rl} W_{\mathrm{BD},m}^{+} & =\frac{N-m}{dm+N-m}\cdot \frac{rm}{rm+N-m-1}\\ \;\text{and}\;\;W_{\mathrm{DB},m}^{-} & =\frac{dm}{dm+N-m}\cdot \frac{N-m}{rm+N-m-r}, \end{array}\right\}$$

and thus,

4.11 $$\gamma_{m}^{\mathrm{DB}}=\frac{d}{r}\times \left(1+\frac{r-1}{rm+N-m-r}\right).$$

Comparing equations (4.6) and (4.11), one can see a duality between the two models which comes naturally from the definition of the models

4.12 $$\gamma_{m}^{\mathrm{DB}}(r,d)=\frac{1}{\gamma_{m}^{\mathrm{BD}}(d,r)}.$$

This results in an interesting connection between the DB and BD results for fixation probability.

Also, the fixation probability is obtained by

4.13 $$\pi_{1}^{\mathrm{DB}}=\frac{(N-1){\left(d-r\right)}^{2}}{{\left(d/r\right)}^{N}(Ndr^{2}-Nr^{3}-dr^{2}+r^{2})+r(r(N-1)+d(r-N))}.$$

For *r*=1 this reduces to equation (4.4),

4.14 $$\frac{d-1}{d^{N}-1}.$$

The expansion of equation (4.13) is

4.15 $$\pi_{1}^{\mathrm{DB}}=1-\frac{d}{r}+\left(\frac{1}{r}-1\right)\cdot \frac{d}{N}+O\left(\frac{1}{N^{2}}\right).$$

Again, the same result can be obtained by first expanding the expression for $\gamma_{m}^{\mathrm{DB}}$, equation (4.11), in terms of 1/*N*, and then performing the summation of a geometric series.

### 4.4 Conclusions for the complete graph scenario

— In the case where ‘self’ is a part of the neighbourhood graph, the BD and DB processes are identical, and we obtain the exact formula for the probability of fixation, equation (4.4).
— If ‘self’ is not included, then the BD and DB processes are different from each other. The difference is of the order of 1/*N*.
— If *d*=1 for the BD process, then the probability of fixation is given by expression (4.4). Similarly, if *r*=1 in the DB process, equation (4.4) holds. These results can be interpreted as the isothermal theorem for the mass action scenario.
— For large values of *N*, the probabilities of fixation can be approximated by expressions (4.9) and (4.15) for the BD and DB processes, respectively.

## 5. Exact results for fixation probability in one dimension

In this section, we focus on the solutions of the DB and BD models on a circle with arbitrary birth and death rates *r* and *d* for the mutant cells. Similar but more restricted cases have been discussed in the literature. Several papers [7,14,24] considered a representation of the model which is a weak-selection limit of the model we discussed in the previous section and showed that the result agrees with the isothermal theorem. Komarova [17] has approached the problem more directly by solving a backward Kolmogorov equation with periodic boundary conditions. We will consider a similar implementation to Komarova and suggest an alternative and more intuitive method to calculate the fixation probability that can be extended to higher dimensions and different connectivities.

**Figure 1.**
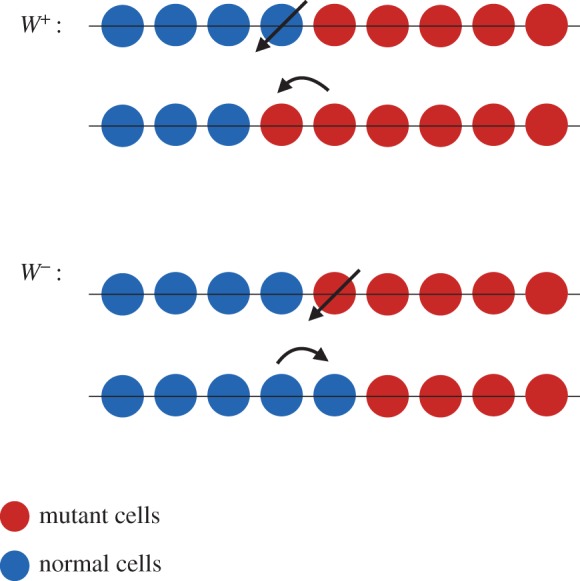
Sequence of events in a DB process on a line (circle). The transition probabilities *W*^±^ are indicated by the corresponding events.

**Figure 2.**
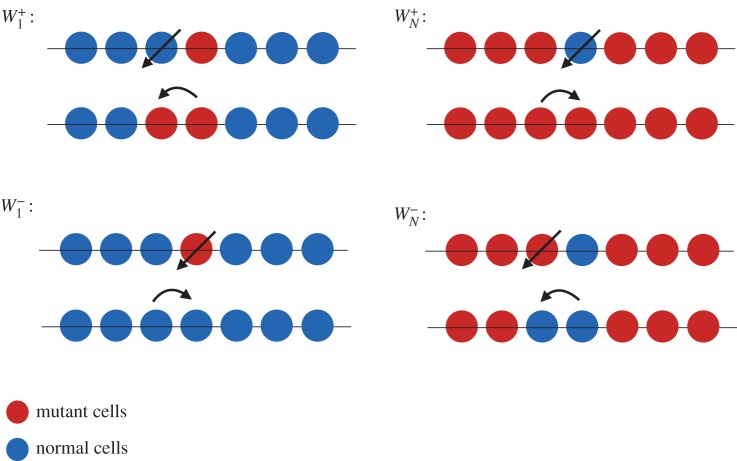
Transition probabilities for the first and last events of fixation. In the text *γ*_1_=*W*^−^_1_/*W*^+^_1_ and *γ*_*N*−1_=*W*^−^_*N*−1_/*W*^+^_*N*−1_. (Figure is depicted for a DB process.)

One dimension is a particular case for which the mutant clone keeps a constant number of normal (or mutant) neighbours. Moreover, the DB events which lead to an increase or decrease of the mutant population only occur at the two ends of the boundary between mutant and normal clones. On the boundary, at any time, the number of mutant neighbours and normal neighbours remain the same. The sequence of events is depicted in figure 1. There are two exceptions to this condition: when there is one mutant cell and *N*−1 normal cells in the system, or *N*−1 mutant cells and 1 normal cell, in which the mutant (normal) cell has two normal (mutant), adjacent neighbouring cells (figure 2). This is not true in higher dimensions. In higher dimensions, the clonal front geometry and even the topology of a mutant clone can fluctuate.

In the following subsections, we will consider the DB and BD processes where the neighbourhoods do *not* include ‘self’. The case where ‘self’ is included is considered in appendix A.

### 5.1 The death–birth model

For the one-dimensional DB model, it turns out that the condition $W_{m}^{-}/W_{m}^{+}=d/r$ holds true for 1<*m*<*N*−1, since in this case the transition probabilities *W*^±^ can be rewritten as

5.1 $$\left.\begin{array}{rl} W_{\mathrm{DB}-1D,m}^{+} & =\frac{1}{m+(N-m)d}\frac{2r}{r+1}\\ \;\text{and}\;\;W_{\mathrm{DB}-1D,m}^{-} & =\frac{d}{m+(N-m)d}\frac{2}{r+1}. \end{array}\right\}$$

The ratio of transition probabilities is different for *m*=1, i.e. where there is only one mutant in the population, and for *m*=*N*−1, where only one normal cell is left in the population. This is due to the fact that the condition of having the same number of mutant or normal cell neighbours on the boundary is not correct in the latter two cases. A single mutant cell in the beginning has no neighbouring mutant cells, and the last remaining normal cell before a full takeover does not have any normal neighbours left. Therefore, we have

5.2 $$\frac{W_{\mathrm{DB}-1D,m}^{-}}{W_{\mathrm{DB}-1D,m}^{+}}=\left\{\begin{array}{ll} \frac{d}{r} & 1<m<N-1\\ \frac{d(r+1)}{2r}\equiv \gamma_{1} & m=1\\ \frac{2d}{\left(r+1\right)}\equiv \gamma_{N-1} & m=N-1. \end{array}\right.$$

Substituting these values into equation (4.2) gives

5.3 $$\pi_{1}^{\mathrm{DB}-1D}=\frac{2d(r-d)}{{\left(d/r\right)}^{N}r^{2}(r-1-2d)+d(d(r-1)+2r)}.$$

In the case where *d*=1, we obtain

$$\pi_{1}^{\mathrm{DB}-1D}=\frac{2(r-1)}{3r-1+(r-3){\left(1/r\right)}^{N-2}}.$$

In the limit N→∞ and *r*>1, this reduces to

$$\pi_{1}^{\mathrm{DB}-1D}=\frac{2(r-1)}{3r-1},$$

a result obtained by Komarova [17]. For *r*=1, equation (5.3) gives

$$\pi_{1}^{\mathrm{DB}-1D}=\frac{1-d}{1-d^{N}},$$

which coincides with equation (4.4) with *r*=1. Equation (5.3) can be also obtained using a generating function method (appendix C).

### 5.2 The birth–death process

Owing to duality between the formulation of a DB process and its corresponding BD process (see equations (3.1) and (3.2)) the same calculation can be repeated for a BD process by switching death and birth rates (d↔r and $W^{+}↔W^{-}$). The transition probabilities for the corresponding BD process are

5.4 $$\frac{W_{\mathrm{BD}-1D,m}^{-}}{W_{\mathrm{BD}-1D,m}^{+}}=\left\{\begin{array}{ll} \frac{d}{r} & 1<m<N-1\\ \frac{2d}{r(d+1)}\equiv \gamma_{1} & m=1\\ \frac{\left(d+1\right)}{\left(2r\right)}\equiv \gamma_{N-1} & m=N-1. \end{array}\right.$$

Substituting these values into equation (4.2) gives

5.5 $$\pi_{1}^{\mathrm{BD}-1D}=\frac{d^{2}(1+d)(d-r)}{d^{2}(d(d-1)-r(d+1))+{\left(d/r\right)}^{N}r(d(r+d+1)-r)}.$$

In the limit where *d*=1, the fixation probability above takes the well-known form for a Moran process

5.6 $$\pi^{\mathrm{BD}-1D}=\frac{1-1/r}{1-{\left(1/r\right)}^{N}},$$

see equation (4.4) with *d*=1.

### 5.3 Comparison of one-dimensional death–birth and birth–death processes

Below we analyse the fixation probabilities for the one-dimensional DB and BD processes obtained in this section, see equations (5.3) and (5.5).

#### 5.3.1 Comparison between the one-dimensional spatial processes and the non-spatial Moran process

**Figure 3.**
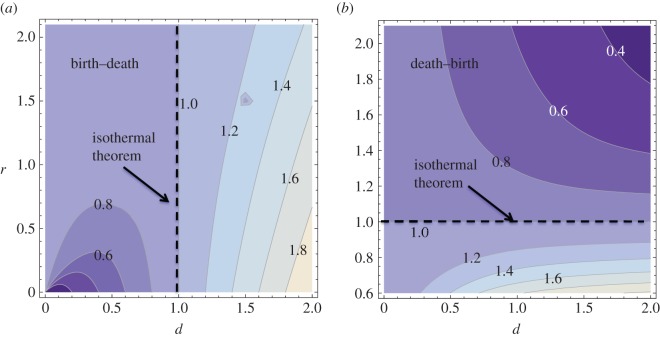
One-dimensional process: comparison of the BD and DB processes with the non-spatial process. (*a*) The ratio *π*^BD−1D^/*π*^Moran^ is plotted as a function of variables *d* and *r*. The contours correspond to equal values of this quantity, and lighter colours mark higher values. (*b*) The same, for the function *π*^DB−1D^/*π*^Moran^. The parameter *N*=100 is used.

We can compare the DB and DB processes with the non-spatial Moran process for general values of *r* and *d*. The results are as follows:

— If *d*>1, the BD process has a higher probability of fixation than the Moran process; if *d*<1, then it has a lower probability of fixation (figure 3*a*).
— If *r*>1, the DB process has a lower probability of fixation than the Moran process; if *r*<1, then it has a higher probability of fixation (figure 3*b*).

In figure 3, we plot the ratio of the probability of fixation in the spatial process (BD in (*a*) and DB in (*b*)) and the non-spatial Moran process. The set of values that satisfy the isothermal theorem separates the regions where the spatial process yields a higher and a lower probability of fixation, compared with the Moran process.

To prove the results above, we consider the cases *d*<*r* and *r*<*d* separately, and take the limit N→∞. For the case of the BD process, the expression *π*^BD−1D^/*π*^Moran^ simplifies to

$$\frac{r+dr}{d-d^{2}+r+dr}\;\text{and}\;\frac{d^{2}(1+d)}{d(1+d+r)-r}$$

in the two cases. Both expressions are greater than 1 if *d*>1, and smaller than one otherwise.

Similarly, for the DB process, in the cases where *d*<*r* and *r*<*d*, the ratio *π*^DB−1D^/*π*^Moran^ simplifies to

$$\frac{2r}{d-r(2+d)}\;\text{and}\;\frac{2d}{r(1+2d-r)}.$$

Both expressions are greater than one if *r*<1, and they are smaller than one otherwise.

The isothermal theorem can be considered a ‘borderline’ case, see the dashed lines in figure 3. This can be summarized by the following statements:

— The BD process satisfies the isothermal theorem in the particular case where *d*=1.
— The DB process satisfies the isothermal theorem if *r*=1.

#### 5.3.2 Comparing the birth–death and death–birth processes to each other

For the same values of *d* and *r*, the DB and BD processes yield different probabilities of fixation. In the special case where *d*=1, the BD process has a higher fixation probability than the DB process iff *r*>1. That is, in the BD process, advantageous mutants have a higher probability of fixation, and disadvantageous mutants have a lower probability of fixation.

If *r*>*d* and N→∞, the probabilities of fixation have a simpler form:

$$\pi^{\mathrm{BD}-1D}=\frac{\left(1+d)(d-r\right)}{d(d-1)-r(d+1)}\;\mathrm{and}\;\pi^{\mathrm{DB}-1D}=\frac{2(r-d)}{d(r-1)+2r}.$$

In this limit, the two processes have the same probability of fixation if

$$r=\frac{3-d}{1+d}.$$

**Figure 4.**
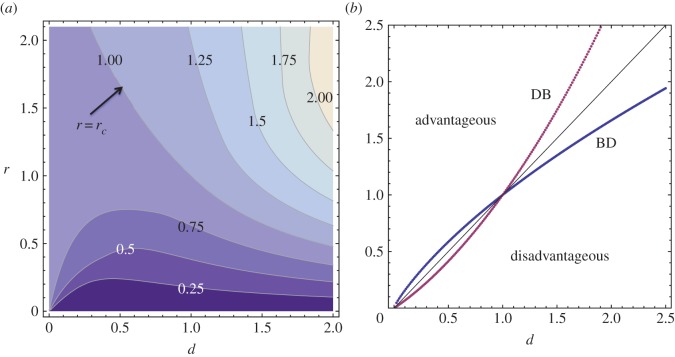
One-dimensional process: properties of the fixation probabilities. (*a*) The ratio *π*^BD−1D^/*π*^DB−1D^ is plotted as a function of variables *d* and *r*. The contours correspond to equal values of this quantity, and lighter colours mark higher values. Above the line *r*=*r*_*c*_, the probability of fixation is larger for the BD process. The parameter *N*=100 is used. (*b*) Neutrality of mutants. Plotted are the lines in the (*d*,*r*) space along which the probability of mutant fixation is equal to 1/*N* (the line *π*^BD−1D^=1/*N* is blue, and the line *π*^DB−1D^=1/*N* is red; the line *r*/*d*=1 is black). Parameter *N*=5 was used.

In general, there is a certain threshold value, *r*_*c*_, such that for *r*>*r*_*c*_ (*r*<*r*_*c*_), the BD process (the DB process) has a higher probability of fixation. This can be interpreted as follows: for mutants that are sufficiently advantageous, the BD process is more successful, and for less advantageous mutants, the DB process is more successful. This is illustrated in figure 4*a*, where we plot the ratio *π*^BD−1D^/*π*^DB−1D^ as a function of the variables *d* and *r*. The contours correspond to equal values of this quantity, and lighter colours mark higher values. Above the line *r*=*r*_*c*_, the probability of fixation is larger for the BD process.

Another interesting limit is for large-*N* and large-*r*. Even in the simple case of *d*=1, while the BD process leads to $\pi_{1}^{\mathrm{BD}}=1-1/r\to 1$, the DB result is $\pi_{1}^{\mathrm{DB}}=2(r-1)/(3r-1)\to \frac{2}{3}$. The fact that in a DB process the fixation probability of an arbitrarily advantageous mutant never approaches unity might sound surprising, but in fact is very intuitive. In a DB updating scheme, no matter how high the chances are for a single advantageous mutant to proliferate, the chance of extinction is always contributed by the early death events. In the case of a one-dimensional cycle, there is a 1/*N* chance for the mutant to be picked and replaced by one of its neighbours in the first time step, while there is 2/*N* chance for the mutant's neighbours to be chosen to die and be replaced by a mutant offspring. The ratio of the total number of events that the population changes to the ones that the mutant survives is $\frac{2}{3}$, which is exactly the fixation probability for r→∞ limit.

#### 5.3.3 Neutrality of the mutants

The spatial processes require a different definition of neutrality for the mutant. In the non-spatial Moran process, neutral mutants satisfy *r*/*d*=1, and the probability of fixation of such mutants is 1/*N*. For one-dimensional spatial models, the sets of neutrality in the space (*d*,*r*) are presented in figure 4*b*. The red line corresponds to the DB process, the blue line to the BD process and the black line to the non-spatial Moran model. These lines become very close to the *r*/*d*=1 line as *N* increases.

## 6. Approximate results for fixation probability in two dimensions

In the following, we apply some of the understanding gained from the previous analysis on DB models and generalize it to find an approximate analytical result in higher dimensions. The one-dimensional case was rather special due to the following properties:

— The number of mutant/normal neighbours at every time during the evolution of the clone remains constant on the two fronts of the mutant clone. The only two exceptions are when there is only one mutant in the system (*N*−1 normal cell) or one normal (*N*−1 mutant).
— Owing to the above property, the ratio of transition probabilities for any clone size remains the same as for the mixed population Moran model other than the two exception of *n*=1 and *n*=*N*−1. Including these two new transition probabilities into the solutions of the Kolmogorov equation leads to an exact result for the fixation probability of the one-dimensional model.
— The topology of the mutant clone does not change with time. If we begin with a single mutant the domain of the mutant clone always remains a simply connected region.

**Figure 5.**
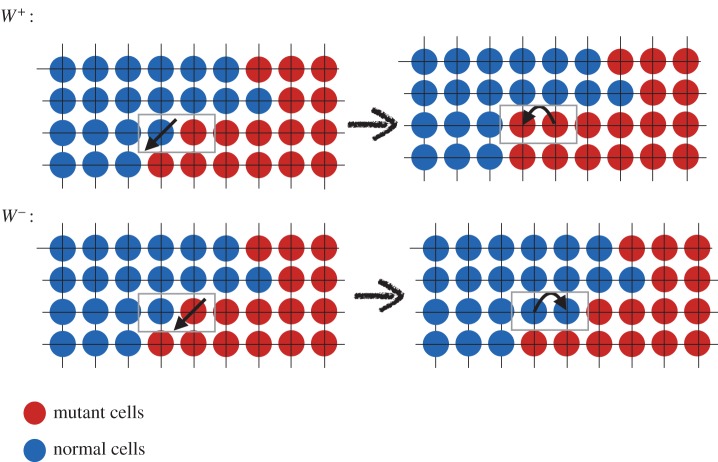
Sequence of death and birth events on the boundary of mutant clone (red) that give rise to the increase or decrease of mutant population. (Figure is depicted for a DB process.)

**Figure 6.**
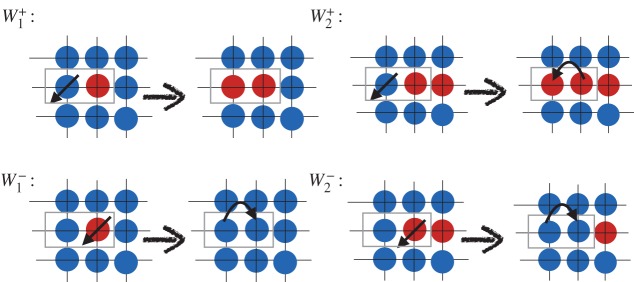
Sequence of death and birth events in the early stages of one-mutant and two-mutant clone (red=mutant). (Figure is depicted for a DB process.)

In higher dimensions, none of the above properties are correct in a strict mathematical sense. The transition probabilities in two or higher dimensions are not only functions of the clone size but of the geometry of the clonal front. Also there are elementary events that can lead to splitting of a clone into two and thus the topology of a clone might change with time. As in the one-dimensional case, we assume periodic boundary conditions and without loss of generality we consider a regular square lattice with degree of connectivity *k*. The sequence of events for a DB process on a square lattice is depicted in figures 5 and 6.

The ratio of transition probabilities in higher dimensions for loss or gain of a mutant cell differs for large clone sizes and small clone sizes. Here, we *assume* that the dominant contribution comes from *m*=1 (in one dimension, *m*=1 was the only different term), and use the approximation $W_{m}^{-}/W_{m}^{+}\approx d/r$ for *m*≥2 (see appendix B). We have

6.1 $$\frac{W_{\mathrm{DB}-2D,m}^{-}}{W_{\mathrm{DB}-2D,m}^{+}}=\left\{\begin{array}{ll} \approx \frac{d}{r} & 2<m<N-2\\ \frac{d(r+k-1)}{\left(kr\right)}=\gamma_{1}^{\mathrm{DB}-2D} & m=1\\ =\frac{d}{r} & m=2. \end{array}\right.$$

Thus, we obtain,

6.2 $$\begin{array}{rl} \pi_{1}^{\mathrm{DB}-2D} & \approx \frac{1}{1+\sum_{j=2}^{N-1}(1+\prod_{k=2}^{j}(W_{k}^{-}/W_{k}^{+}))\gamma_{1}^{\mathrm{DB}-2D}},\\ & =\frac{1}{1+((1-{\left(d/r\right)}^{N-2})/(1-(d/r)))\gamma_{1}^{\mathrm{DB}-2D}}, \end{array}$$

and upon substitution from equation (6.1) we end up with an algebraic expression for $\pi_{1}^{\mathrm{DB}-2D}$, for large *N*,

6.3 $$\pi_{1}^{\mathrm{DB}-2D}\approx \frac{k(r-d)}{kr+d(r-1)}.$$

**Figure 7.**
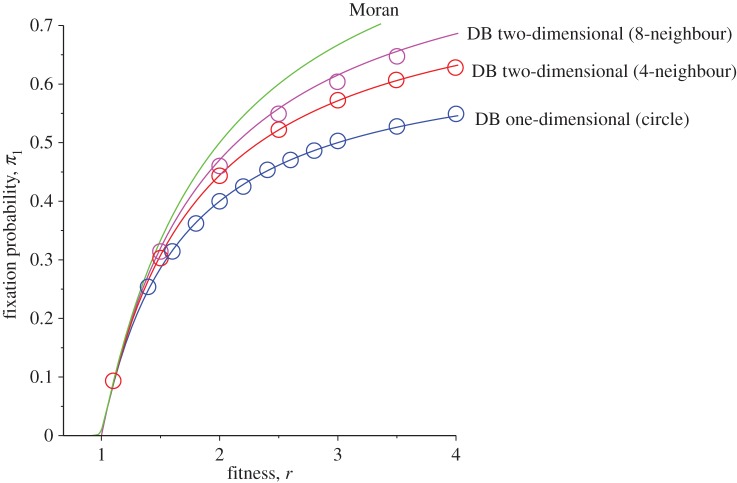
Comparison of analytical results for the fixation probability *π*_1_ as a function of the division rate *r* for one-dimensional DB (*N*=20) and two-dimensional DB (*N*=20×20) cases with *k*=4 (4-neighbour) and *k*=8 (8-neighbour) regular lattices with periodic boundary conditions. The solid lines are analytical results from equation (4.7) (mixed population Moran model), equation (5.3) (one-dimensional DB with *d*=1) and equation (6.3) (two-dimensional DB with *d*=1), while circles are the results of stochastic simulations with 40 000 iterations.

For *d*=1 and *k*=2, this reduces to 2(*r*−1)/(3*r*−1). For large *k*, we obtain *π*^DB−2D^_1_≈1−*d*/*r*. Equation (6.3) can be compared with the result of the stochastic simulations result on a square lattice (*k*=4, *d*=1) as shown in figure 7.

**Figure 8.**
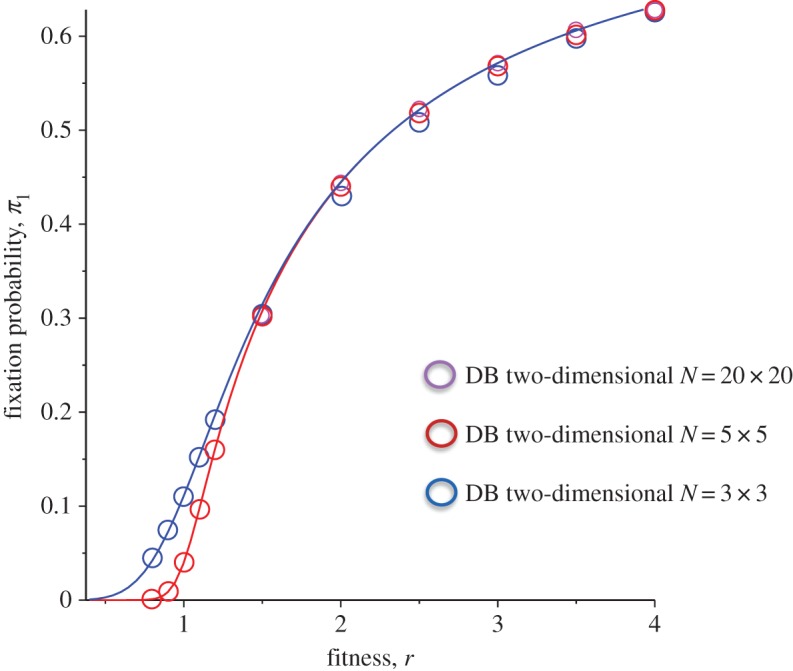
Comparison of analytical results for the fixation probability *π*_1_ as a function of the division rate *r* for a square lattice two-dimensional DB with different lattice sizes cases with *k*=4 (4-neighbour). Solid lines are analytical results from equation (6.2), while circles are the results of stochastic simulations with 40 000–80 000 iterations (for smaller sizes, we used higher number of iterations to reduce the statistical error).

We can even test the validity of this approximation for two-dimensional regular graphs for smaller lattice sizes. To do so, we can use the finite-*N* contribution from equation (6.2). In figure 8, we plotted the result for square lattices (*k*=4) with *N*=3×3 and *N*=5×5 and *N*=20×20. The match is good for closer to neutral limit cases while there is slight deviation from the analytical prediction as fitness increases away from the neutral limit.

**Figure 9.**
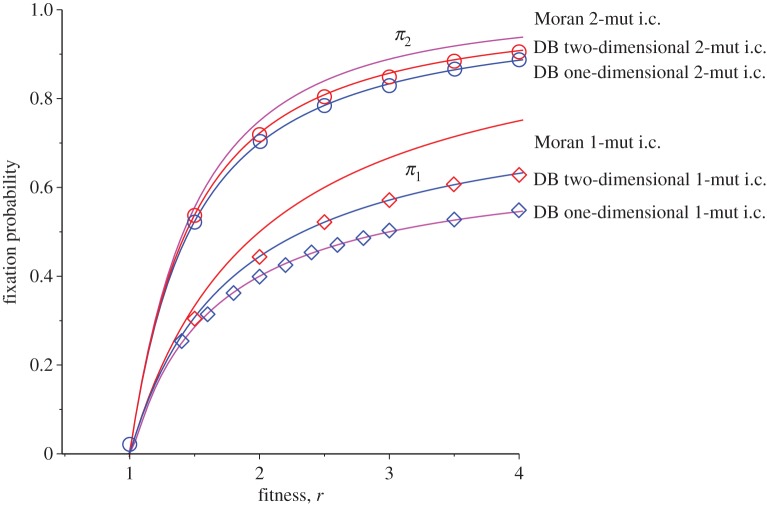
Comparison of fixation probability for two-mutant and one-mutant initial conditions for *N*=100 (one-dimensional DB) and *N*=20×20 square lattice (two-dimensional DB) as a function of division rate *r*. Solid lines are corresponding theoretical results. As can be seen the two-mutant results are closer to Moran formula, equation (6.5).

We can also further test validity of equations (6.2) and (6.3) by comparing the results for fixation probability when one begins with *two* mutants, i.e. the transition probability *γ*_1_ which is claimed to be the main cause of deviation from isothermal behaviour in the DB case does not have any effect and we do expect the fixation probability to be close to a mixed population formula with the two-mutant initial condition. Note that *γ*_1_ is not the only cause of deviation from the isothermal result as other transition probabilities *γ*_*i*_ are only approximately *l*/*r*. This is in fact the case for the results of one- and two-mutant fixation probabilities which are depicted for a square lattice with *N*=20×20 and *d*=1 in figure 9.

Recalling the Moran fixation probability with the two-mutant initial condition *π*_2_

6.4 $$\pi_{2}^{\mathrm{Moran}}=\frac{1-{\left(1/r\right)}^{2}}{1-{\left(1/r\right)}^{N}}.$$

The exact result can be obtained (in either one-dimensional circle or two-dimensional regular lattices) by using the recursive relation

6.5 $$\pi_{2}=\left(1+\frac{1}{r}\right)\pi_{1},$$

which is obtained from solutions of the Kolmogorov equation. This leads to the following results for *π*_2_ in one- and two-dimensional cases in the large-*N* limit,

6.6 $$\left.\begin{array}{rl} \pi_{2}^{\mathrm{DB}-1D} & =\frac{3r+1}{3r-1}\left(1-\frac{1}{r}\right)\\ \;\text{and}\;\;\pi_{2}^{\mathrm{DB}-2D} & =\frac{5r+3}{5r-1}\left(1-\frac{1}{r}\right). \end{array}\right\}$$

The above approximation fits large-*N* simulation results well as shown in figure 9. Since equations (6.6) are obtained using a mixed population form of the Kolmogorov equation, the fits with the simulations further support our assumptions in deriving the formula for the two-dimensional DB fixation probability, given by equation (6.2).

In the case of a BD updating, while the special case of *d*=1 leads to the isothermal theorem for regular graphs, the fixation probability for arbitrary values of *r* and *d* might not follow isothermal behaviour analogous to the case discussed earlier. Similar to the two-dimensional DB case we obtain

6.7 $$\frac{W_{\mathrm{BD}-2D,m}^{-}}{W_{\mathrm{BD}-2D,m}^{+}}=\left\{\begin{array}{ll} \approx \frac{d}{r} & 2<m<N-2\\ \frac{kd}{\left(r(d+k-1)\right)}=\gamma_{1}^{\mathrm{BD}-2D} & m=1\\ =\frac{d}{r} & m=2. \end{array}\right.$$

This gives, for large *N*,

6.8 $$\pi_{1}^{\mathrm{BD}-2D}\approx \frac{\left(d+k-1)(r-d\right)}{(d+k-1)(r-d)+kd}.$$

For *d*=1, equation (6.8) reduces to 1−1/*r* (independent of *k*). For large *k*, we have *π*^BD−2D^_1_≈1−*d*/*r*. Setting *k*=2, this reduces to the one-dimensional case.

## 7. Discussion

In this work, we discussed generalized versions of DB and BD updating models for evolutionary dynamics on graphs and spatial structures. In contrast to most previous approaches, we assume that the mutants can differ from the wild-type not only by their birth rate (*r*), but also by their death rate (*d*), giving rise to a two-parameter model where *r* and *d* are independent arbitrary parameters affecting the selection process. This is in fact more realistic, as in many scenarios either death rate or birth rate or both can vary and affect the selection dynamics in tissues in situations involving invasion mechanisms such as cancer. Focusing on regular lattices with periodic boundary conditions, we show that the models for both DB and BD updating are exactly solvable in a one-dimensional circle.

We further studied to what extent the isothermal theorem can be generalized. This theorem states that the selection dynamics are the same on any isothermal graph as they are for a mixed population/complete graph. This theorem was proved by Lieberman *et al.* [5] to hold for the *d*=1 BD update, and here we show that it also holds for the *r*=1 DB update. We further demonstrated that the more general, two-parameter models deviate from this theorem. The BD and DB systems exhibit fixation probabilities that are different from each other and from the canonical mass-action Moran result.

While for the case of the complete graph the difference is vanishingly small for large graphs, it remains finite for one- and two-dimensional spatial structures. In general, we conjecture that the difference between the two models has the order of the inverse of the degree of connectivity of the graph. For example, the connectivity of the complete graphs (the mass-action system) is *N*, and thus the difference scales with 1/*N*. The connectivity of regular spatial lattices is given by the number of neighbours of each node. In the one-dimensional ring, the number of neighbours is 2, and in typical two-dimensional lattices, it is 4 or 8. Thus, the difference in the fixation probability between the BD and DB models is the largest for the one-dimensional ‘nearest-neighbour’ ring, it is slightly smaller in the case of the von Neumann (4-cell) neighbourhood in two dimensions, and still smaller in the case of the Moore (8-cells) neighbourhood. It decays with the number of neighbours.

The reason for the deviation from the isothermal theorem is the different transition probabilities for the case when there is only one mutant in the system, compared to the rest of the transition probabilities. When the quantity of interest is the fixation probability starting from more than one mutant cells, then the difference between DB and BD models (and the deviation from the isothermal theorem) is smaller, compared with the case when we start with one mutant.

Depending on the parameters, the spatial DB and DB processes could be characterized by a smaller or larger mutant fixation probability compared with the conventional non-spatial Moran result. In particular, for the BD process, the fixation probability is higher than the Moran value as long as *d*>1. For the DB process, the fixation probability is higher than the Moran value as long as *r*<1.

The generalized two-parametric BD and DB models can be applied to investigate the evolutionary dynamics of tissue turnover. We would like to emphasize the importance of considering both birth and death rates of mutants (compared to those of the surrounding cells) in terms of the conceptual construct of the hallmarks of cancer [34]. Three of them are the most relevant for our study. They are listed below and put in our context by linking them with the cells' birth and death rates [35–37]:

1. *Self-sufficiency in growth signals*. While normal cells cannot proliferate in the absence of stimulatory signals, cancer cells can do this with the help of oncogenes, which mimic normal growth signalling. This can be achieved by means of different mechanisms. For example, cells can acquire the ability to synthesize their own growth factors, e.g. the production of PDGF (platelet-derived growth factor) and TGF-*α* (tumour growth factor *α*) by glioblastomas and sarcomas. Furthermore, cell surface receptors that transduce growth-stimulatory signals into the cell, for example, EGFR and HER2/neu, may be overexpressed or structurally altered, leading to ligand-independent signalling. Finally, downstream targets of the signalling pathway can be altered, e.g. the Ras oncogene, which is found mutated in about 25% of human tumours. In all these cases, the mutant cells are characterized by an *increased birth rate* compared with the surrounding cells.
2. *Insensitivity to antigrowth signals*. Antigrowth signals can block proliferation by (i) forcing cells out of the active proliferative cycle into the quiescent (G0) state, until appropriate growth signals put them back into the cell cycle or (ii) inducing differentiation, which permanently removes their proliferative potential. Cancer cells evade these antiproliferative signals, by e.g. loss of TGF-*β*, loss of Smad4, or loss of CDK inhibitors such as p16, p21 or p53. The corresponding cells again are characterized by an *increased birth rate* compared with the surrounding cells.
3. *Evading apoptosis*. The ability of tumour cell populations to expand in number is determined not only by the rate of cell proliferation but also by the rate of cell attrition. Programmed cell death (apoptosis) represents a major source of this attrition. Resistance to apoptosis can be acquired by cancer cells by e.g. loss of p53 (which normally activates pro-apoptotic proteins and represents the most common loss of a pro-apoptotic regulator), or by activation or upregulation of anti-apoptotic Bcl2. In these cases, the mutants are characterized by a *decreased death rate* compared with the surrounding cells.

Other applications are the cases when selection dynamics are affected by the introduction of a drug which increases the mutant cell death while the birth rate is determined by the cell division rate and is independent of the drug concentration. This can be applied to problems in both cancer and infectious diseases, where spatial structures affect selection dynamics.

Another interesting application is the stem cell dynamics in the case of the intestinal crypt. Recently, Vermuelen *et al.* [38] investigated the dynamics of niche success in the intestinal crypt base by inducing different types of mutations, including APC, p53 and Kras, and were able to measure the fraction of mutant stem cell clones at various time points for a wide number of crypts (in mice). The authors fitted the results to a simple BD model on a one-dimensional ring. They used Bayesian inference to infer model parameters such as proliferation rate *r* and total niche size *N*. It has been observed that Kras oncogenic mutation infers a relatively high selection advantage in a newly introduced mutant to the stem cell niche while APC^−/+^ is weakly disadvantageous and APC^−/−^ mutation in a background of APC^−/+^ mutants is weakly advantageous. Similarly, it has been reported that p53 mutations in a normal intestinal base infer a very small selection advantage while in the inflamed gut the advantageous p53 has the higher chance of succeeding in the niche.

**Figure 10.**
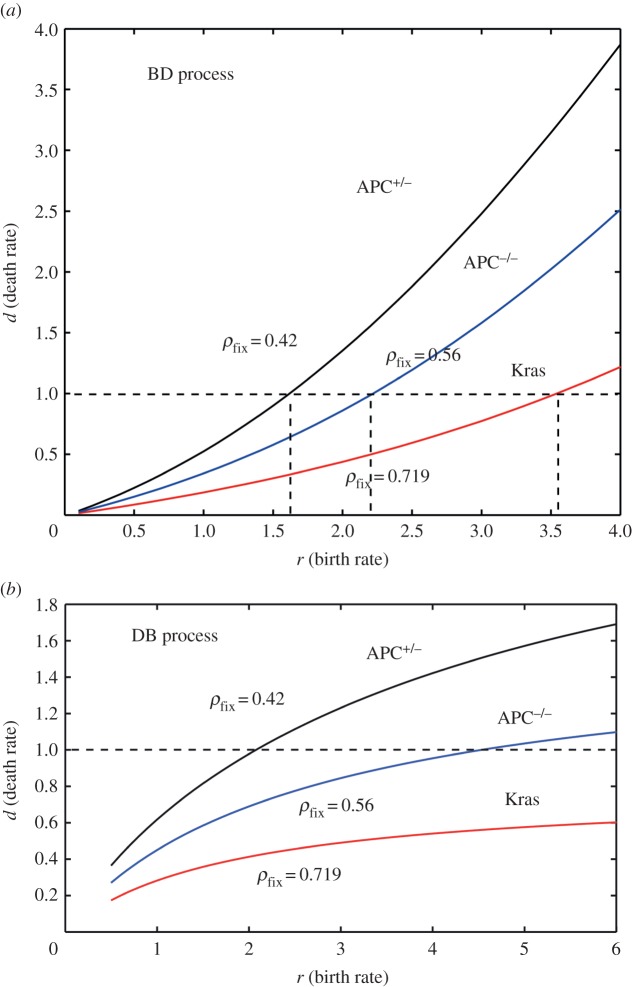
The plots of (*d*,*r*) sets for different mutations. The values of the fixation probability are extracted from Vermeulen *et al.* [38].

As discussed above, to compare with the experimental data, the birth and death rates of normal and mutant stem cells should be taken into account independently, particularly when both mechanisms are contributing to the selection dynamics at the same time. In the case of intestinal crypt stem cells, aside from the fact that different mutations can confer different death and birth rates (or combination of both), other mechanisms such as symmetric differentiation, and cell cycle and quiescent states can act as additional effective mechanisms for a death event in such an evolutionary model. We have used the reported fixation probability to estimate the possible set of death and birth rates. We also applied both DB and BD processes to see whether there is any significant difference between the two models. The results are depicted in figure 10*a*,*b*.

In figure 10*a*, we have plotted the lines indicating possible sets of birth rate and death rate for three mutants, Kras, APC^−/+^ and APC^−/−^. Dashed lines indicate the values for death rates *d*=1 and various birth rates *r* reported in Vermeulen *et al.* [38]. Similar results have been depicted for the case of a DB process where the death event occurs first. In the case of relatively advantageous mutations, such as Kras, the *d*=1 case leads to an unusual high division rate. In fact, higher divisor rates also point to much higher birth rates due to negative curvature of the (*r*,*d*) graphs. This is basically a special case of the duality reported in the previous sections. Our finding also supports the belief that the stem cell dynamics inside a crypt niche is dominated by birth (division) events, followed by death events due to geometrical constraints in the systems. Such death events some times are referred to as a ‘retraction’ in the biological context [39].

## Appendix A. One-dimensional processes where ‘self’ is included in the neighbourhood network

Let us consider a one-dimensional, nearest-neighbour process, where the cell itself is considered to be one of its own neighbours. Therefore, instead of two neighbours, each cell has three neighbours. Performing calculations similar to those of §5, we obtain the following results. Note that although qualitatively the fixation probabilities behave similar to those of §5, the numerical values differ significantly in the two processes. Thus, whether or not we include ‘self’ in the neighbourhood will significantly change the resulting fixation probabilities, and the change does not disappear in the large *N* case, like it did in the mass-action scenario.

**A.1. The birth–death process**

We have the following expression for transition rate ratios:

A 1 $$\gamma_{m}^{\mathrm{BD}}=\frac{W_{\mathrm{BD},m}^{-}}{W_{\mathrm{BD},m}^{+}}=\left\{\begin{array}{ll} \frac{d(1+2d)}{\left(r(d+2)\right)} & 1<m<N-1\\ \frac{d}{r} & m=1\\ \frac{d}{r} & m=N-1 \end{array}\right.$$

Note that if *d*=1, we have $\gamma_{m}^{\mathrm{BD}}=1/r$ for all *m*. Using equation (4.2), the probability of fixation can be easily calculated. Because the expression is rather cumbersome, here we only present the approximation for the case when, simultaneously, *r*>*d*, *r*>*d*(1+2*d*)/(2+*d*), and N→∞:

$$\pi_{1}^{\mathrm{BD}}=1-\frac{d(d+2)}{r(d+2)-d(d-1)}.$$

The isothermal theorem (for the general *N*) is satisfied for *d*=1.

**A.2. The death–birth process**

In this case, the transition rate ratios become

A 2 $$\gamma_{m}^{\mathrm{DB}}=\frac{W_{\mathrm{DB},m}^{-}}{W_{\mathrm{BD},m}^{+}}=\left\{\begin{array}{ll} \frac{d(r+2)}{\left(1+2r\right)} & 1<m<N-1\\ \frac{d}{r} & m=1\\ \frac{d}{r} & m=N-1. \end{array}\right.$$

If *r*=1, we have $\gamma_{m}^{\mathrm{DB}}=d$ for all *m*. Using equation (4.2), the probability of fixation can be calculated; again, the expression is cumbersome. Here we only present the approximation for the case when, simultaneously, *r*>*d*, *d*<*r*(1+2*r*)/(*r*+2), and N→∞:

$$\pi_{1}^{\mathrm{BD}}=1-\frac{d(1+2r)}{d(r-1)+r(2r+1)}.$$

The isothermal theorem is satisfied for *r*=1.

## Appendix B. Two-dimensional graph transition probability

In this section, we briefly discuss the ratio of transition probabilities for both DB and BD models when the mutant population is large. For simplicity, we assume *d*_A_=*d*_B_=1, *r*_A_=1 and *r*_B_=*r*. Using equations (3.2), the ratio of transition probabilities are given by

B 1 $$\frac{W_{\mathrm{BD}}^{-}}{W_{\mathrm{BD}}^{+}}=\frac{\sum_{i,j}(w_{ji}n_{j}(1-n_{i})/((r-1)\sum_{k}n_{k}+N))}{\sum_{i,j}(rw_{ji}n_{i}(1-n_{j})/((r-1)\sum_{k}n_{k}+N))}.$$

For isothermal graphs the sum over indices *i* and *j* can be switched and the ratio becomes 1/*r* for any configuration. On the other hand, this is not quite true for the DB process. The ratio of transition probabilities using equations (3.1) are

B 2 $$\frac{W_{\mathrm{DB}}^{-}}{W_{\mathrm{DB}}^{+}}=\frac{\sum_{i,j}(w_{ij}n_{i}(1-n_{j})/N((r-1)\sum_{l}w_{il}n_{l}+k))}{\sum_{i,j}(rw_{ij}n_{j}(1-n_{i})/(N((r-1)\sum_{l}w_{il}n_{l}+k)))},$$

where $k=\sum_{l}w_{il}$ is the number of neighbours for each site. Note that the denominator and numerator do not simplify as the terms in the denominator now depend on *i* as well. However, for large mutant populations, we can approximate $(r-1)\sum_{l}w_{il}n_{l}+k$ and make it independent of the index *i*. In this case, one can simplify the ratio and arrive at the result 1/*r*. This is, however, incorrect for smaller populations, as discussed in §6. The main contribution comes from the *m*=1 population. For *m*=1,2 (*m* is the mutant population), we have

B 3 $$\left.\begin{array}{rl} \frac{W_{1}^{-}}{W_{1}^{+}} & =\frac{r+3}{4r}\\ \;\text{and}\;\;\frac{W_{2}^{-}}{W_{2}^{+}} & =\frac{6/(r+3)}{6r/(r+3)}=\frac{1}{r}. \end{array}\right\}$$

Similar calculations for *m*=3 gives $W_{3}^{-}/W_{3}^{+}\approx 1/r$.

## Appendix C. Generating function formalism

**C.1. Mixed population in weak selection**

In the following, we repeat the alternative derivation of the fixation probability for a non-spatial Moran model from Houchmandzadeh & Vallade [40]. Beginning with the master equation for the Moran process in the weak-selection limit

C 1 $$\begin{array}{r} \frac{\partial p(n;t)}{\partial t}=W^{+}(n-1)p(n-1;t)+W^{-}(n+1)p(n+1;t)-(W^{+}(n)+W^{-}(n))p(n,t), \end{array}$$

where *p*(*n*;*t*) is the probability of having *n* mutants at time *t* with a given initial condition. The transition rates *W*^±^(*n*) are given by

C 2 $$\left.\begin{array}{rl} W^{+}(n) & =\frac{r\cdot n(N-n)}{\left((rn+(N-n))(dn+(N-n)\right)}\\ \;\text{and}\;\;W^{-}(n) & =\frac{d\cdot n(N-n)}{\left((rn+(N-n))(dn+(N-n)\right)}, \end{array}\right\}$$

where the transition probabilities at the absorbing states are taken to be zero, *W*^±^(*n*=*N*)=*W*^±^(*n*=0)=0. Houchmandzadeh & Vallade [40] considered the special case of the above model where *d*=1, *r*−1≪1. This way the denominator in transition probabilities *W*^±^(*n*) can be approximated by *N*^2^. The probability generating function *F*(*z*,*t*):

C 3 $$F(z,t)=\sum_{m=0}z^{n}p(m,t),$$

obeys the following equation (*d*=1):

C 4 $$\frac{\partial F(z,t)}{\partial t}=(z-1)(rz-1)\frac{\partial }{\partial z}\left(N\cdot F(z,t)-z\frac{\partial F(z,t)}{\partial z}\right).$$

Note that equation (C 4) is derived using the identity

C 5 $$\sum_{n}nz^{n}=z\frac{\partial }{\partial z}\sum_{n}z^{n}.$$

Boundary and initial conditions for the PDE in equation (C 4) are

C 6 $$\left.\begin{array}{rl} F(1,t) & =1\;(\mathrm{boundary}\;\mathrm{condition})\\ \;\text{and}\;\;F(z,t=0) & =z^{n_{0}}\;(\mathrm{initial}\;\mathrm{condition}), \end{array}\right\}$$

where *n*_0_ is the number of mutant cells at *t*=0. The steady-state solution for *F*(*z*,*t*) is

C 7 $$F(z,t\to \infty )=A+Bz^{N},$$

where coefficients A and B are extinction and fixation probabilities. Applying the boundary and initial conditions and noticing that *z**=1/*r* is a fixed point of equation (C 4), we can solve for the coefficient B to obtain

C 8 $$\pi_{n_{0}}=B=\frac{1-{\left(1/r\right)}^{n_{0}}}{1-{\left(1/r\right)}^{N}}.$$

The trivial fixed point *z**=1 leads to the absorption probability being unity. This corresponds to the initial condition of beginning with *N* initial mutants. Note that, in general, we do not need to find a fixed point of the PDE for the generating function but rather a point *z** (or set of points) for which the right-hand side of equation (C 4) is zero.

**C.2. Evolutionary graph models in weak selection**

The above method can be generalized to derive the fixation probability of the DB (BD) model on a graph. This method has been developed by Houchmanzadeh and Vallade in the weak selection limit of BD and DB processes [7,41]. We will generalize their method to account for exact solutions for arbitrary values of *r* and *d* in one dimension.

Consider a DB model with transition probabilities

C 9 $$\left.\begin{array}{rl} W_{\mathrm{DB}}^{+}(n_{i}) & \equiv P(n_{1},\ldots ,n_{i},\ldots ,n_{N}\to n_{1},\ldots ,n_{i}+1,\ldots ,n_{N})\\ & =\frac{1-n_{i}}{(d-1)\sum_{l}n_{l}+N}\frac{r\sum_{j}w_{ij}n_{j}}{(r-1)\sum_{k}w_{ik}n_{k}+1}\\ \;\text{and}\;\;W_{\mathrm{DB}}^{-}(n_{i}) & \equiv P(n_{1},\ldots ,n_{i},\ldots ,n_{N}\to n_{1},\ldots ,n_{i}-1,\ldots ,n_{N})\\ & =\frac{dn_{i}}{(d-1)\sum_{l}n_{l}+N}\frac{\sum_{j}w_{ij}(1-n_{j})}{(r-1)\sum_{k}w_{ik}n_{k}+1}, \end{array}\right\}$$

and master equation corresponding to the above transition probabilities is

C 10 $$\begin{array}{r} \frac{\partial p(\{n_{i}\},t)}{\partial t}=W^{+}(n_{i}-1)p(n_{i}-1,t)+W^{-}(n_{i}+1)p(n_{i}+1,t)-(W^{+}(n_{i})+W^{-}(n_{i}))p(n_{i},t), \end{array}$$

where *p*(*n*_*i*_±1;*t*)≡*p*(*n*_1_,…,*n*_*i*_±1,…,*n*_*N*_). Introducing the probability generating function *F*({*z*_*i*_};*t*):

C 11 $$F(z_{1},\ldots ,z_{N};t)\equiv \sum_{\{n_{k}\}=0,1}z_{0}^{n_{0}}z_{1}^{n_{1}}\cdots z_{N}^{n_{N}}p(n_{1},\ldots ,n_{N};t),$$

and using the master equation (C 10), an equation for the generating function can be obtained (see [7] and also previous section),

C 12 $$\begin{array}{rl} \frac{\partial F(\{z_{k}\},t)}{\partial t} & ={\hat{N}}_{d}^{-1}\sum_{i}(z_{i}-1){\hat{N}}_{r,i}^{-1}\sum_{j}rw_{ij}\left(1-z_{i}\frac{\partial }{\partial z_{i}}\right)z_{j}\frac{\partial }{\partial z_{j}}F\\ & \;+{\hat{N}}_{d}^{-1}\sum_{i}(z_{i}^{-1}-1){\hat{N}}_{r,i}^{-1}\sum_{j}dw_{ij}\left(1-z_{j}\frac{\partial }{\partial z_{j}}\right)z_{i}\frac{\partial }{\partial z_{i}}F, \end{array}$$

where operators ${\hat{N}}_{d}^{-1}$ and ${\hat{N}}_{r}^{-1}$, are inverse of ${\hat{N}}_{d}$ and ${\hat{N}}_{r}$, given by

C 13 $$\left.\begin{array}{rl} {\hat{N}}_{d} & =(d-1)\sum_{k}z_{k}\frac{\partial }{\partial z_{k}}+N\\ \;\text{and}\;\;{\hat{N}}_{r,i} & =(r-1)\sum_{k}w_{k,i}z_{k}\frac{\partial }{\partial z_{k}}+1. \end{array}\right\}$$

Similar to the mixed population case, boundary and initial conditions for the spatial case are

C 14 $$\left.\begin{array}{rl} F(z_{1}=1,\ldots ,z_{N}=1,t) & =1\;(\mathrm{boundary}\;\mathrm{condition})\\ \;\text{and}\;\;F(z_{1},\ldots ,z_{N},t=0) & =z_{1}^{n_{1}^{0}}\cdots z_{N}^{n_{N}^{0}}\;(\mathrm{initial}\;\mathrm{condition}). \end{array}\right\}$$

Here, $n_{i}^{0}$ is the number of mutant cells at each site *i* at *t*=0. The system has only two absorbing state corresponding to fixation or extinction of the mutants introduced to the system indicating the following stationary form of the generating function (see [7] for detailed discussion):

C 15 $$F(\{z_{k}\},t\to \infty )=A+Bz_{0}z_{1}\cdots z_{N},$$

where A and B are constants to be determined from the boundary and initial conditions, respectively. The fixed points of equation (C 12) indicated by $\left\{z_{1}^{*},\ldots ,z_{N}^{*}\right\}$ determine the fixation probability [7]

C 16 $$\pi_{1}=B=\frac{1-F(z_{1}^{*},\ldots ,z_{N}^{*};t)}{1-z_{1}^{*}z_{2}^{*}\cdots z_{N}^{*}}.$$

Given the initial condition and assuming that $n_{1}^{0}=1$, and $n_{i}^{0}=0,\;i\neq 1$, the fixation probability simplifies to:

C 17 $$\pi_{1}=B=\frac{1-z_{1}^{*}}{1-z_{1}^{*}z_{2}^{*}\cdots z_{N}^{*}}.$$

To find *z**'s in this limit, we put the terms in front of first derivative and second derivatives (∂/∂*z*_*i*_) terms equal to zero, separately. In the limit of *d*=1 and *r*≪1 both these operators are proportional to unity and can be dropped from the rest of the calculations in the same manner as in the previous section for mixed population. The first-order derivative term gives rise to a nonlinear relationship between *z*_*i*_'s,

C 18 $$\sum_{j}w_{ji}(1-z_{j}^{*})=\frac{d}{r}\left(1-\frac{1}{z_{i}^{*}}\right),$$

where the second-order terms cancel out for $z_{i}^{*}=d/r$. It is straightforward to check that equation (C 18) is satisfied for *z*_*i*_=*d*/*r* in an isothermal graph. In the next part, we discuss the one-dimensional limit that also give rise to exact solutions for equation (C 12).

**C.3. One-dimensional solutions**

In the case of a one-dimensional circle, the operator *N*_r,*i*_ takes a simple form assuming that we begin with a single mutant at site *i*=1 at *t*=0 and additionally assuming that the last normal cell to be replaced by a mutant was at some site indexed by *i*=*K*. This leads to the following form for *N*_r,*i*_:

C 19 $$N_{r,i}=\left\{\begin{array}{ll} 2 & i=1\\ 2r & i=K\\ \left(r+1\right) & i\neq 1,\;i\neq K.\; \end{array}\right.$$

This significantly simplifies equation (C 12) and solutions for $z_{i}^{*}$ that makes the RHS of equation (C 12) zero can be obtained (in a similar manner equation (C 18) was derived). Assuming $\left\{z_{k}^{*}\}=\{z_{1}^{*},z_{2}^{*}=z^{*},\ldots ,z_{K-1}^{*}=z^{*},z_{K}^{*},z_{K+1}^{*}=z^{*}\ldots ,z_{N}^{*}=z^{*}\right\}$, the equation for $z_{1}^{*}$ and $z_{K}^{*}$ can be written in terms of all other fixed points, while $z_{i}^{*}=d/r,\;i\neq \{1,K\}$,

C 20 $$\left.\begin{array}{rl} & \sum_{j}\frac{r}{r+1}(z_{j}-1)w_{ij}-\frac{d}{2}\left(\frac{1}{z_{1}}-1\right)=0\\ \;\text{and}\;\; & \sum_{j}\frac{d}{r+1}\left(\frac{1}{z_{j}}-1\right)w_{ij}-\frac{r}{2r}(z_{K}-1)=0,\;j\neq 1,K, \end{array}\right\}$$

which can be further simplified to

C 21 $$\left.\begin{array}{rl} \frac{2r}{d(r+1)}(z^{*}-1) & =1-\frac{1}{z_{1}^{*}}\\ \;\text{and}\;\;\frac{r+1}{2d}(z_{K}^{*}-1) & =1-\frac{1}{z^{*}},\;z^{*}=\frac{d}{r}, \end{array}\right\}$$

leading to,

C 22 $$\left.\begin{array}{rl} z_{1} & =\frac{(r+1)d}{(r-1)d+2r},\\ z_{K} & =\frac{2d-r+1)}{r+1}\\ \;\text{and}\;\;z_{i} & =\frac{d}{r}\;i\neq \{1,K\}. \end{array}\right\}$$

Substituting the above values of fixed points into equation (C 17),

C 23 $$\begin{array}{rl} \pi_{1}^{1D-\mathrm{DB}} & =\frac{1-z_{1}^{*}}{1-z_{1}^{*}z_{K}^{*}{\left(z^{*}\right)}^{N-2}},\\ & =\frac{2d(r-d)}{d(d(r-1)+2r)+{\left(d/r\right)}^{N}(r^{2}(r-1-2d))}, \end{array}$$

which is exactly the results for the one-dimensional DB model obtained in §4. An exactly similar calculation can be repeated to obtain the one-dimensional BD fixation probability as well.

Similarly, it can be argued that for a square lattice, in the large-*N* limit, equation (C 20) leads to (by including appropriate *N*_r_ for two-dimensional case, and for *d*=1)

C 24 $$\frac{4r}{r+3}(z^{*}-1)=1-\frac{1}{z_{0}^{*}},$$

leading to the result for the fixation probability given by equation (6.3), with *k*=4,

C 25 $$\pi_{1}^{2D}=1-z_{1}^{*,2D}=\frac{4(r-1)}{5r-1}.$$
